# The Reorganization of Insular Subregions in Individuals with Below-Level Neuropathic Pain following Incomplete Spinal Cord Injury

**DOI:** 10.1155/2020/2796571

**Published:** 2020-03-10

**Authors:** Xuejing Li, Ling Wang, Qian Chen, Yongsheng Hu, Jubao Du, Xin Chen, Weimin Zheng, Jie Lu, Nan Chen

**Affiliations:** ^1^Department of Radiology, Xuanwu Hospital, Capital Medical University, 100053, China; ^2^Beijing Key Laboratory of Magnetic Resonance Imaging and Brain Informatics, 100053, China; ^3^Department of Radiology, Beijing Friendship Hospital, Capital Medical University, 100050, China; ^4^Department of Functional Neurosurgery, Xuanwu Hospital, Capital Medical University, 100053, China; ^5^Department of Rehabilitation Medicine, Xuanwu Hospital, Capital Medical University, 100053, China; ^6^Department of Radiology, Aerospace Central Hospital, 100049, China

## Abstract

**Objective:**

To investigate the reorganization of insular subregions in individuals suffering from neuropathic pain (NP) after incomplete spinal cord injury (ISCI) and further to disclose the underlying mechanism of NP.

**Method:**

The 3D high-resolution T1-weighted structural images and resting-state functional magnetic resonance imaging (rs-fMRI) of all individuals were obtained using a 3.0 Tesla MRI system. A comparative analysis of structure and function connectivity (FC) with insular subareas as seeds in 10 ISCI individuals with below-level NP (ISCI-P), 11 ISCI individuals without NP (ISCI-N), and 25 healthy controls (HCs) was conducted. Associations between the structural and functional alteration of insula subregions and visual analog scale (VAS) scores were analyzed using the Pearson correlation in SPSS 20.

**Results:**

Compared with ISCI-N patients, when the left posterior insula as the seed, ISCI-P showed increased FC in right cerebellum VIIb and cerebellum VIII, Brodmann 37 (BA 37). When the left ventral anterior insula as the seed, ISCI-P indicated enhanced FC in right BA18 compared with ISCI-N patients. These increased FCs positively correlated with VAS scores. Relative to HCs, ISCI-P presented increased FC in the left hippocampus when the left dorsal anterior insula was determined as the seed. There was no statistical difference in the volume of insula subregions among the three groups.

**Conclusion:**

Our study indicated that distinctive patterns of FC in each subregion of insula suggest that the insular subareas participate in the NP processing through different FC following ISCI. Further, insula subregions could serve as a therapeutic target for NP following ISCI.

## 1. Introduction

Individuals with spinal cord injury (SCI) often have varying degrees of neuropathic pain (NP), which seriously affects the quality of life and functional rehabilitation [1, 2]. At present, there is no effective treatment for NP secondary to SCI because of the lack of understanding of the underlying mechanism. Some studies suggest that the sensitization of central and peripheral nervous system and associated cortical remodeling are considered main reasons in the development of NP following SCI [3–5]. These reorganizations located in areas associated with nociceptive processing [4, 6, 7], such as primary sensory cortex (S1), secondary somatosensory cortex (S2), and thalamus. However, other studies about NP following SCI have not found any changes in these areas [8, 9]. Therefore, NP management may be involved in the complex connections associated with the key node of pain network rather than simply structural changes [10].

Recently, the insula, as a part of limbic system, has been gradually recognized as a vulnerable region in the condition of NP [11, 12]. There exists the D1 and D2 dopamine receptors in the insula [13, 14], which participates in the modulation of chronic nociception [15, 16]. Another regulatory component is the *γ*-aminobutyric acid (GABA) receptor, which acts mainly to modulate the nociceptive threshold [17]. Morphological studies have found the changes of insular volume in individuals with NP, such as reduced insular volume in SCI individuals with NP [5, 18]. And the ventral anterior insula (v-AI) volume would be normalized after effective treatment in patients with trigeminal neuralgia (TN) [19]. Furthermore, the increased insular cerebral blood flow (CBF) was highly correlated with pain intensity [20, 21]. Therefore, the changes in insular morphology and function are inevitably associated with pain after SCI, while the anterior and posterior of insula differ in cytoarchitecture and function [22, 23], the subregions of insula have been implicated in the different functional profiles in the perception of pain [11, 24, 25]. The anterior insula (AI) principally receives afferent from the medial nuclei of the thalamus [26] and is interconnected with the ventrolateral prefrontal and orbitofrontal cortex, which is associated with the cognitive-affective components of nociceptive processing [11]. The posterior insula (PI) is a part of the cortical network mainly involved in the sensory regulation of pain through the connectivity with S1, S2, and the midcingulate cortex [11, 27]. The PI also participates in the maintenance of persistent pain [28] and is implicated in the process of chronic pain [29]. However, earlier studies about the role of insular subareas in the regulation of pain have mainly focused on healthy controls (HCs) or rodent animals [10, 11, 30], and few studies have paid attention to the functions of insula subregion in the pathological pain. Considering the high prevalence of NP secondary to SCI and the insular susceptibility and heterogeneity in NP state, it is essential to reveal the reorganization of the insular subareas in incomplete SCI (ISCI) patients with NP. In the current study, we examined whether and how the structure and function of insular subregions altered following ISCI, aiming to systematically investigate the underlying mechanism of NP.

## 2. Materials and Methods

### 2.1. Participants

A total of 46 individuals in three groups were recruited, including 10 ISCI individuals with below-level NP (ISCI-P) (male 7 and female 3, with a mean age of 50.7 ± 13.8 years), 11 ISCI individuals without NP (ISCI-N) (male 4 and female 7, with a mean age of 49.4 ± 13.7 years), and 25 HCs (14 men and 11 women, with a mean age of 53.2 ± 7.0 years). All the subjects met the inclusion criteria: right-handedness, no preexisting mental illness or cognitive disorders affecting the functional outcome, and without history of associated brain diseases confirmed by conventional MRI. Exclusion criteria were MRI contraindications, poor image quality, traumatic brain injury, presence of a systemic medical illness, or central nervous system disorder.

The study protocol was approved by the Ethics Committee of Xuanwu Hospital of Capital Medical University, Beijing, China. Written informed consent in accordance with the Declaration of Helsinki was obtained from each participant of this study.

### 2.2. Clinical Assessment

Before magnetic resonance imaging (MRI), all individuals were interviewed to determine the existence of pain using European Multicenter Study about SCI pain questionnaire (V4.2, http://www.emsci.org/). The pain questionnaire examines various aspects of pain (e.g., duration (years), maximal, and average pain intensity). To be classified as below-level NP, ongoing pain had to be located three of more segments below the level of lesion [31]. The pain intensity was rated using an 11-point numeric rating scale (visual analog scale, VAS) with “0” indicating no pain to “10” indicating worst pain imaginable. All individuals with SCI underwent a comprehensive clinical assessment. Briefly, motor, sensory, and neurological levels of injury were identified allowing characterization of sensory/motor functioning as well as determination of the completeness of injury according to the International Standards for Neurological Classification of Spinal Cord Injury Impairment Scale [32]. The detailed information of patients was provided in Table 1.

### 2.3. Imaging Data Acquisition

MRI data were acquired by a Siemens 3.0 Tesla MR system. Routine brain axial fluid-attenuated inverse recovery (FLAIR) sequence scanning was first performed to rule out other cerebral abnormalities. Sagittal three-dimensional T1-weighted images were acquired by the following scan parameters: repetition time (TR) = 1800 ms; echo time (TE) = 2.13 ms; inversion time (TI) = 1100 ms; flip angle (FA) = 9°; field of view (FOV) = 256 mm × 256 mm; matrix = 256 × 256; slice thickness = 1 mm, no gap; and 192 sagittal slices. The resting-state functional data were acquired using a gradient-echo-planer imaging (EPI) sequence with the following parameters: TR = 2000 ms, TE = 30 ms, slice thickness = 3 mm, number of slices = 35, FOV = 220 mm × 220 mm, matrix size = 64 × 64, interslice gap = 0.8 mm, FA = 9°. As for this section, we followed the methods of Chen et al. [33].

### 2.4. Definition of Insular Subregions

**Figure 1:**
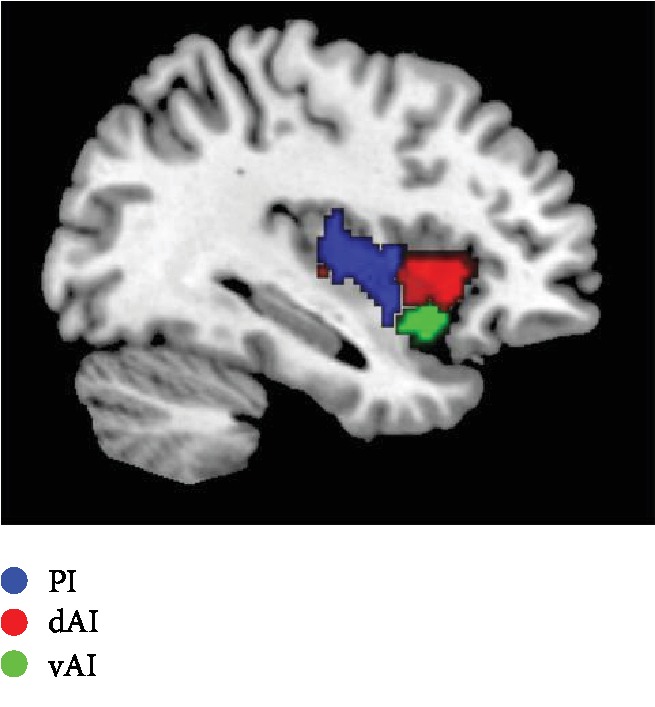
Sagittal view displaying the insula subregions.

The insula was divided into three subregions in each hemisphere, based on a connection-based parcellation study, in which the insula was subdivided according to whole brain FC, through data-driven clustering technique [34]. The cluster analysis identified 3 insular subregions in each hemisphere: the posterior insula (PI, blue), the dorsal anterior insula (dAI, red), and the ventral anterior insula (vAI, green) (Figure 1).

### 2.5. Structure Data Preprocessing and Analysis

First, the raw DICOM images were reviewed and converted into the Neuroimaging Informatics Technology Initiative format, using MRICRON software. Next, we performed the preprocessing steps using the CAT12 toolboxes with the default setting. Briefly, in CAT12 toolboxes, all 3D T1-weighted MRI scans are normalized using an affine followed by nonlinear registration, corrected for bias field in homogeneities, and then segmented into gray matter (GM), white matter (WM), and cerebrospinal fluid (CSF) components. For both procedures, we used the Diffeomorphic Anatomical Registration Through Exponentiated Lie (DARTEL) algebra algorithm to normalize the segmented scans into a standard MNI space. Next, the normalized GM and WM images were smoothed using a Gaussian kernel of 8 mm full width at half maximum (FWHM). In the CAT12 toolboxes, the total GM volume (GMV), WM volume, and CSF volume were obtained, on the basis of segmented images. The total intracranial volume (TIV) was calculated as the sum of the GM, WM, and CSF volumes for each toolbox, separately. To further identify the morphological abnormalities, the insula subregions were defined as the seed points. Next, the mean volume values of each insula subregions of every subject were extracted. Then we compared the GMV differences in each insula subregions between ISCI-P, ISCI-N, and HCs based on SPSS version 20.0 (IBM, Armonk, NY, USA). Bonferroni correction was employed in multiple comparisons (*P* < 0.05). Post-hoc analysis was employed to determine GMV differences in group pairs (*P* < 0.05, Bonferroni correction).

### 2.6. fMRI Data Preprocessing and Analysis

The resting-state fMRI data were preprocessed with Data Processing & Analysis for (Resting-State) Brain Imaging (DPABI) (http://www.rfmri.org/dpabi). The first 10 volumes of the time series were discarded to accommodate for fluctuations induced while longitudinal magnetization became stabilized. Then, images were slice-timing corrected, realigned, and resliced to correct for head motion with a mean volume created. Head motion between volumes was evaluated and corrected using rigid body registration. And all subjects' fMRI data were within defined motion thresholds (maximum translation or rotation less than 2.0 mm or 2.0°). Then, the corrected fMRI images were spatially normalized to the MNI template brain based on the standard stereotaxic coordinate system. Next, each voxel was resampled to isotropic 3 mm × 3 mm × 3 mm. Twenty-six nuisance covariates were regressed to remove possible variances from the time course of each voxel, including WM and CSF signals, as well as Friston 24 head motion parameters. After normalization, all data sets were smoothed with a Gaussian kernel of 8 × 8 × 8 FWHM. As for fMRI data preprocessing, we followed the methods of Chen et al. [33]. The insula subregions were defined as seed points for the following FC analysis. After band-pass filtering (0.01–0.08 Hz) and linear trend removal, a reference time series for each seed was extracted by averaging the time series of voxels within each insula subregions. Finally, correlation analysis was performed between the seed region and the remaining voxels in the whole brain. To improve normality, the resulting *r* values were converted by Fisher's *r*-to-*z* transformation [35]. The GLM with age, sex as covariates, was used to analyze FC differences among ISCI-P, ISCI-N, and HCs using one-way ANOVA analysis based on SPM12 (a voxel-level uncorrected *P* < 0.0001, cluster-level family-wise error correction with *P* < 0.05). Post-hoc analysis was employed to determine FC differences in group pairs (*P* < 0.05, FWE correction).

### 2.7. Statistical Analysis

All data were analyzed for normality and homogeneity of variance. One-way ANOVA and post-hoc analysis were employed for data with normal distribution. Data identified as not normally distributed were assessed by nonparametric tests. Partial correlation analysis was performed to explore any potential associations between the alteration of insula subregion's structure and function and VAS scores in ISCI patients after removing gender and age effects based on SPSS version 20.0 (IBM, Armonk, NY, USA).

## 3. Results

### 3.1. Clinical Data

In the final analysis, three participants (including 2 HCs and 1 ISCI-N) were excluded on the basis of the criterion of head motion of more than 2 mm in any direction or an angular rotation greater than 2°. Comparisons across the three subject groups showed no significant differences in gender (Kruskal-Wallis, *P* = 0.376) and age (one-way ANOVA, *P* = 0.204). All the 21 SCI individuals were incomplete injuries, as measured by the American Spinal Cord Injury Association (ASIA) scale (http://www.asiaspinalinjury.org/). Among of these individuals, 10 patients suffered from chronic below-level NP (the duration of pain > 1 year) [36]. The mean pain intensities were 7.4 ± 1.8. The remaining 11 individuals with SCI were pain-free at the time points of measurements.

### 3.2. Morphological Changes of Insula Subregions between ISCI-P, ISCI-N, and HCs

There was no structural difference in insula between groups. As for the morphological changes in insula subregions, compared with HCs, ISCI-P patients showed decreased GMV, whereas ISCI-N showed increased GMV. However, these differences were not statistically significant. The detailed information was shown in Table 2.

### 3.3. Altered Function Connectivity of Insular Subregions between ISCI-P, ISCI-N, and HCs

**Figure 2:**
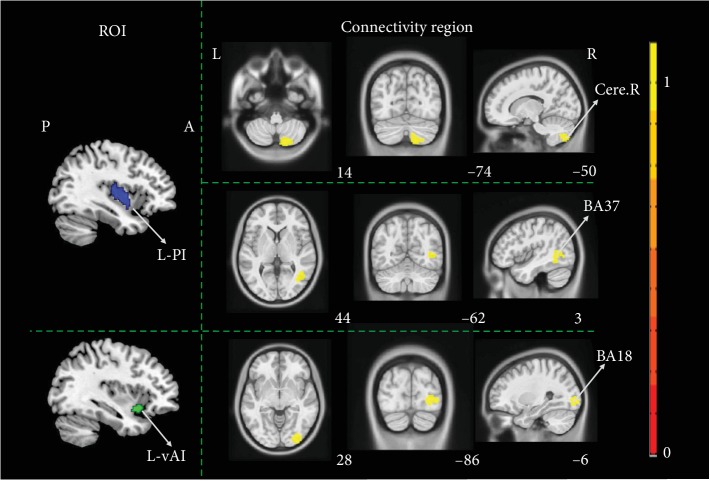
The altered function connectivity of insula subregions between ISCI-P and ISCI-N. Compared with ISCI-N patients, ISCI-P showed increased FC in right cerebellum VIIb and VIII, BA37 when the L-PI as the seed point, and enhanced FC in BA18 when the L-vAI was chosen as the ROI (cluster level, family-wise error (FWE) *P* < 0.05). ISCI-P: incomplete spinal cord injury with neuropathic pain; ISCI-N: incomplete spinal cord injury without neuropathic pain; FC: function connectivity; BA: Brodmann; L-PI: left posterior insula; L-vAI: left ventral anterior insula; ROI: region of interest; Cere.R: right cerebellum; P: posterior; A: anterior; L: left; R: right. Hot color represents 1‐*P* value.

Compared with ISCI-N patients, ISCI-P showed increased function connectivity (FC) in right cerebellum VIIb and VIII, Brodmann 37 (BA 37) when the L-PI was selected as the seed point, and enhanced FC in BA18 when the L-vAI was chosen as the ROI (cluster-level FWE correction with *P* < 0.05) (Figure 2).

**Figure 3:**
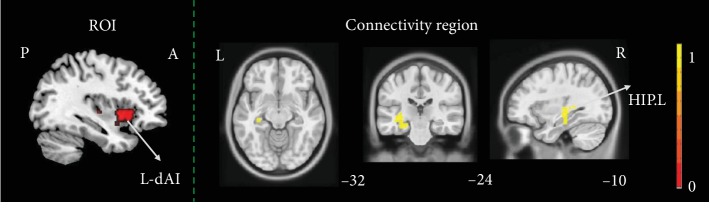
The altered function connectivity of insula subregions between ISCI-P and HCs. Relative to HCs, ISCI-P presented increased FC in left hippocampus gyrus when the L-dAI as seed point (cluster level, family-wise error (FWE) *P* < 0.05). ISCI-P: incomplete spinal cord injury with neuropathic pain; HCs: healthy controls; FC: function connectivity; P: posterior; A: anterior; L: left; R: right; L-dAI: left dorsal anterior insula; HIP.L: left hippocampus. Hot color represents 1‐*P* value.

Relative to HCs, ISCI-P presented increased FC in left hippocampus when the L-dAI as seed point (cluster-level, FWE correction with *P* < 0.05) (Figure 3).

There was no difference found between ISCI-N and HCs whichever insula subregions were chosen as seed points. In addition, there was no abnormal FC between the three groups when the seed sites were selected in the right insular subregions.

### 3.4. Associations of VAS Scores and FC Alteration between ISCI-P and ISCI-N

**Figure 4:**
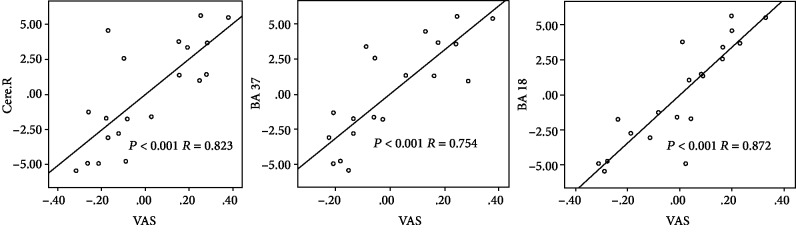
Association of VAS scores and FC alteration between ISCI-P and ISCI-N. Partial correlation analysis revealed a positive correlation between the alteration of FC, including right cerebellum VIIb and VIII (*P* < 0.001, *R* = 0.823), BA37 (*P* < 0.001, *R* = 0.754), BA18 (*P* < 0.001, *R* = 0.872), and the VAS scores. VAS: visual analog scale; FC: function connectivity; ISCI-P: incomplete spinal cord injury with neuropathic pain; ISCI-N: incomplete spinal cord injury without neuropathic pain; Cere.R: right cerebellum; BA: Brodmann.

In the subsequent correlation analysis, the increased FC, including right cerebellum VIIb and VIII (*P* < 0.001, *R* = 0.823), BA37 (*P* < 0.001, *R* = 0.754), and BA18 (*P* < 0.001, *R* = 0.872), between ISCI-P and ISCI-N was positively correlated with the VAS scores (Figure 4).

## 4. Discussion

Our study indicated that distinctive patterns of FC in each subregion of insula suggest that the insular subareas participate in the nociceptive processing through different connectivity following ISCI. Specifically, the left PI is interconnected with the right cerebellum VIIb and VIII and BA37, and the left AI is part of the cortical network mainly involved in the regulation of pain through the FC with BA18 and hippocampus gyrus.

### 4.1. Morphological Changes of Insular Subregions between ISCI-P, ISCI-N, and HCs

Compared with HCs, ISCI-P patients showed decreased GMV in each subregion of insula. Pathologically, the alteration of insula volume was due to neuronal death, synaptic pruning secondary to loss of afferent deprived central areas [37]. Clinically, the insula belongs to the medial pain system and possesses a pivotal role in encoding affective-motivational aspects of pain [38, 39]. Thus, the altered structure of insula may be associated with depression and adverse emotions [19].

Whereas ISCI-N showed increased GMV compared with ISCI-P, the alteration in GM could result from the pathologic formation of FC with the cerebellum, as is confirmed through the following functional part. Apart from, the growth of the insular GM is a dynamic process in the state of NP after SCI [19], just as some studies have found that the abnormalities of insula structure are reversible by significantly increasing with effective treatment, which might reflect the normalization of emotional components or other cognitive functions related to the multidimensional experience of pain [19]. In our present study, although, these differences were not statistically significant, which is most likely due to the small groups. Future research based upon large sample is needed to further study the effect of the insular GM on the progression and prognosis of pain.

### 4.2. Altered Functional Connectivity of Insula Subregions between ISCI-P, ISCI-N, and HCs

Previous studies have demonstrated that there were two isolated pain regulation systems in the human brain, which administers different functional responsibilities [40, 41]. One system is the lateral pain system, which includes S1 and S2, and the lateral thalamic nuclei and is believed to be primarily associated with the process of the sensory discriminative aspects of pain. The other system is the medial pain system, including ACC, insular cortex, prefrontal cortex (PFC), and the medial thalamic nuclei. It is thought to primarily involve the affective-motivational aspects of pain. Accordingly, the insula is traditionally considered an important component of the medial pain system and participates in cognitive and emotional regulation, such as empathy, depression, and disgust [42, 43].

However, recent neuroimaging evidences suggested the disparate functions ascribed to the insula in the perception of pain [25, 44]. In our present FC analysis, we found there was no statistical difference with right insula as seeds. The possible reason is as follows: all the subjects in the present study were right-handed. Whereas, whether symmetrical information transmission between the right and left insula occurs remained to be investigated [18]. In addition, no difference of FC was found between ISCI-N and HCs whichever insula subregions were chosen as seed points, which suggests that these changes observed are pain related not injury related.

ISCI-P patients revealed increased FC in the cerebellum posterior lobe, including right cerebellum VIIb and VIII compared with ISCI-N when the seed sites were located in left PI. Accumulating clinical evidences have demonstrated that the cerebellum played a crucial role in pain perception and modulation in humans [45, 46]. The cerebellum participates in the regulation of pain mainly through three aspects: first, the cerebellum has a wealth of connections with the dorsolateral prefrontal cortex (DLPFC) [47, 48], the brainstem and periaqueductal [49], and these regions are involved in descending pain modulatory pathways [50]. However, animal studies have suggested that activation of the anterior and posterior cerebellum might be anti-nociceptive and pro-nociceptive, respectively [51, 52]. In our current findings, we found the increased activation mainly in the cerebellum posterior lobe, which might increase spinal noxious responses and decrease the latency of withdrawal from nociceptive stimulation [51]. Second, some researchers observed overlapping activation in bilateral cerebellum lobules VI and VIIb, when pain and motor processes were combined within the same experiment [53]. Thus, they proposed that the posterior cerebellum might be vital in pain-associated adaptations in movement [54]. In our present results, we found the increased activation in cerebellum VIIb, which may be beneficial to produce pain-related movement behaviors. Third, a sufficient body of evidence prompts that some noninvasive telencephalon stimulation methods, such as cerebellar transcranial direct current and magnetic stimulation, have emerged as promising and effective techniques for modulating pain experience [46, 55], the cerebellum engagement in both the sensory-discriminative and emotional dimensions of pain [56, 57]. Our results indicated that the enhanced activation mainly in the right cerebellum VIIb and VIII may provide a theoretical basis for precisely selecting stimulus targets. Further, previous researches have indeed corroborated the existence of the pathway between insula and cerebellum [58, 59]. Additionally, we found the positive correlation between the FC changes and VAS scores. Based on all the aspects mentioned above, we speculated that the left PI-cerebellum posterior lobe pathway participates in the modulation of pain perception and intensity following SCI, which is expected to be an exploration of underlying neural mechanisms of NP, and provided a theoretical basis for selection of target of stimulation [52]. We also found the enhancive activation in the BA 37 and BA18. The increased activity in BA37 had been reported during the hyperalgesia [60]. And activation in BA 18 was observed responding to virtual pain stimuli by fMRI [61]. Pain is a multifaceted experience involving the synergy of multiple brain regions. However, the specific mechanism of these brain regions still needs to be disclosed.

ISCI-P presented increased FC in left hippocampus when the left d-AI as seed point. The AI, which is one of the core hubs of salience network (SN) [62], played an important role in the dynamic switching between the task negative network (default mode network, DMN) and task positive networks (executive control network, ECN and sensorimotor network, SMN) [63–65]. Based on the point, the activation of AI may make a positive response to exogenous noxious stimuli by activating the ECN and suppressing the DMN [63]. On the other hand, the limbic system, including the hippocampus gyrus, is activated in pain processing [66]. The hippocampus is possibly involved in the formation of pain-associated memory and emotional responses [67, 68]. Earlier researches have proposed that the sensory and limbic networks of pain are parallel and independent [25]. Follows a nociceptive stimulation, an initial noxious processing in sensory and limbic systems is rapidly followed by a functional integration of both toward the AI. The convergence of multimodal afferent to AI contributes to emotional awareness and the building of multifaceted but unified subjective perception [69]. Further, some researchers found the reduced activations in the hippocampus and the AI following analgesic administration [70], which have an effect on affective component of pain. Our current findings showed the increased FC in left hippocampus gyrus and left d-AI, which may have a positive effect on pain perception.

There are two major limitations. First, only right-handed subjects were recruited. And the researches combined with the left-handed may contribute to the understanding of insular function. Second, the sample size was relatively small. Future research based upon large sample is needed.

## 5. Conclusions

Our observations suggested that the reorganization of insula subregions occurs in the state of NP following ISCI. The insula subregions participate in the regulation of NP through the different FC following ISCI. These findings may contribute to the understanding of NP neural mechanisms and provide an accurate treatment target for NP following ISCI.

## Figures and Tables

**Table 1:** Demographic data and clinical values of the SCI subjects.

Subjects	Age (years)	Gender	Time since injury (years)	Level of lesion	ASIA score	Motor (0–100)	Sensory (0–224)	Neuropathic pain	VAS
1	60	M	3	C3-7	D	70	204	Below-level	9
2	56	M	33	C4	C	9	142	Below-level	8
3	57	M	7	C4	D	90	158	Below-level	6
4	71	M	1	C3-4	D	80	204	Below-level	5
5	34	F	1	L1-3	D	74	190	Below-level	4
6	40	F	12	L1-2	D	96	172	Below-level	8
7	37	M	17	L1	D	95	182	Below-level	8
8	37	F	3	L1	C	58	148	Below-level	8
9	40	M	16	T12	B	50	164	Below-level	9
10	42	M	6	T12	C	54	144	Below-level	9
11	55	F	6	T6-7	D	80	188	No	0
12	68	F	2	T8	D	96	188	No	0
13	53	M	4	C5-6	D	90	176	No	0
14	49	M	1	C2-7	D	90	206	No	0
15	54	F	1	C2-3	D	79	188	No	0
16	24	F	2	C3-7	C	64	212	No	0
17	49	M	5	C3-4	D	95	216	No	0
18	37	M	3	C4-7	D	80	224	No	0
19	31	F	2	C3-5	C	60	212	No	0
20	58	F	2	C4-7	D	90	200	No	0
21	65	F	1	C4-6	D	80	180	N0	0

The level of lesion refers to the neurological level. ASIA impairment scale: A: complete—no sensory or motor function is preserved in sacral segments S4–S5; B: incomplete—sensory but not motor function is preserved below the neurological level and extends through sacral segments S4−S5; C: incomplete—motor function is preserved below the neurological level, and more than half of the key muscles below the neurological level have a muscle grade of <3; D: incomplete—motor function is preserved below the neurological level, and at least half of the key muscles below the neurological level have a muscle grade of >3. Sensory score: sum of segmental light touch and pinprick classifications. ASIA: American Spinal Injury Association. VAS: visual analog scale.

**Table 2:** Intergroup differences of gray matter volume in insula subregions.

Insula-subareas	Groups			*F* value	*P* value
	ISCI-P (mm^3^)	ISCI-N (mm^3^)	HCs (mm^3^)		
L-dAI	1890 ± 0.292	2048 ± 0.352	1963 ± 0.257	0.786	0.462
L-PI	1174 ± 0.191	1234 ± 0.225	1214 ± 0.166	0.283	0.755
L-vAI	769 ± 0.119	833 ± 0.127	807 ± 0.080	1.073	0.351
R-dAI	2109 ± 0.398	2256 ± 0.345	2220 ± 0.270	0.620	0.543
R-PI	932 ± 0.157	1012 ± 0.135	995 ± 0.125	1.047	0.360
R-vAI	913 ± 0.131	968 ± 0.135	944 ± 0.103	0.584	0.562

Results are displayed as mean ± standard deviation. L: left; R: right; dAI: dorsal anterior insula; PI: posterior insula; vAI: ventral anterior insula; ISCI-P: incomplete spinal cord injury with neuropathic pain; ISCI-N: incomplete spinal cord injury without neuropathic pain; HCs: healthy controls.

## Data Availability

The data used to support the findings of this study are available from the corresponding author upon request.
